# A general ecophysiological framework for modelling the impact of pests and pathogens on forest ecosystems.

**DOI:** 10.1111/ele.12345

**Published:** 2014-08-28

**Authors:** Michael C Dietze, Jaclyn Hatala Matthes

**Affiliations:** 1 Department of Earth and Environment, Boston UniversityBoston, Massachusetts; 2 Department of Geography, Dartmouth CollegeHanover, New Hampshire

**Keywords:** Biotic disturbance, ecosystem modelling, forest insects and pathogens, functional types, pathogen and insect pathways

## Abstract

Forest insects and pathogens (FIPs) have enormous impacts on community dynamics, carbon storage and ecosystem services, however, ecosystem modelling of FIPs is limited due to their variability in severity and extent. We present a general framework for modelling FIP disturbances through their impacts on tree ecophysiology. Five pathways are identified as the basis for functional groupings: increases in leaf, stem and root turnover, and reductions in phloem and xylem transport. A simple ecophysiological model was used to explore the sensitivity of forest growth, mortality and ecosystem fluxes to varying outbreak severity. Across all pathways, low infection was associated with growth reduction but limited mortality. Moderate infection led to individual tree mortality, whereas high levels led to stand-level die-offs delayed over multiple years. Delayed mortality is consistent with observations and critical for capturing biophysical, biogeochemical and successional responses. This framework enables novel predictions under present and future global change scenarios.

## Introduction

Forest insects and pathogens (FIPs) are the dominant cause of ecosystem disturbance in many forest ecosystems, and consequently FIPs play a critical role in regulating emergent biogeochemical cycles (Metcalfe *et al.* 2013). Across the United States and Canada, the impact of FIPs is similar in magnitude to that of both fire and forestry combined (Hicke *et al.* 2012). Native FIPs play a fundamental role in maintaining biodiversity, particularly in tropical ecosystems (Janzen 1970; Connell 1971; Bagchi *et al.* 2014). Invasive FIPs have functionally eliminated a number of keystone species, such as American chestnut (*Castanea dentata*) (Anagnostakis 1987; Loo 2008), and can have large detrimental effects on biodiversity, carbon storage and resulting ecosystem services (Peltzer *et al.* 2010; Boyd *et al.* 2013). Wood- and phloem-boring FIPs alone create $1.7 billion per year in costs to local governments in the United States, whereas local residents bear an additional $830 million per year in lost residential property values (Aukema *et al.* 2011). Although the interactions between FIPs and climate change are complex, in many scenarios FIPs are estimated to increase the rate and magnitude of disturbance within forests that experience novel climates (Trãn *et al.* 2007; Weed *et al.* 2013; Thompson *et al.* 2014). The future feedbacks between FIPs and global change represent one of the largest uncertainties in projecting the future carbon sink of forest ecosystems (Kurz *et al.* 2008b).

Observational data suggest that FIPs vary considerably in the pathways, severities and timescales of their impacts. Even within a single FIP species, there can be considerable heterogeneity in the resulting ecosystem impact depending on environmental conditions, dispersal and aggregation responses, and plant stress (Orwig *et al.* 2002; Peltonen *et al.* 2002; Fettig *et al.* 2007; Bright *et al.* 2012). Most FIPs are associated with reductions in tree growth during and after an infection, which can result in mortality ranging in spatial scale from the patchy loss of individual trees to continental-scale die-offs (Hicke *et al.* 2012). A critical difference between disturbances caused by FIPs and those from clear-cuts and fires is that in many cases mortality due to FIPs is not instantaneous; it is often the cumulative result of ongoing stress that can sometimes persist for decades (Hatala *et al.* 2011). This variability in the scale and rate of mortality can have large impacts on land surface biophysics, such as changes in the surface energy budget, hydrology, canopy turbulence and snowpack. Furthermore, the spatiotemporal spectrum of scales and rates of mortality will generate a cascade of impacts on biogeochemical rates and community dynamics that influences the rate and trajectory of forest recovery, habitat structure and biodiversity.

Due to this heterogeneity in the pathways, severities and timescales by which FIPs impact forest ecosystems, they have not been represented in ecosystem models in any general framework (Hicke *et al.* 2012). When the impacts of FIP disturbances are modelled at an ecosystem scale, they tend to be case studies, investigating the impact of a single FIP during a single outbreak (Albani *et al.* 2010; Pfeifer *et al.* 2010; Medvigy *et al.* 2012). In most case studies that model the impact of FIPs, outbreaks are typically implemented as causing immediate tree mortality (Albani *et al.* 2010; Pfeifer *et al.* 2010), and thus may be misestimating the pace of subsequent community, biogeochemical and biophysical responses. Furthermore, because the long-term growth reductions due to FIPs are absent from models, this could lead to the systematic overestimation of carbon uptake and storage across wide regions. Moreover, heterogeneity in the pathways through which FIPs act means that non-lethal impacts are not limited to a reduction in woody growth increment, but can involve changes in plant carbon pool turnover, allocation and water use.

In this manuscript, we propose a general scheme for the representation of FIP disturbance in ecosystem models. Analogous to the division of vegetation into broad Plant Functional Types in ecosystem models, we propose dividing FIPs into broad groups based on how they impact plant ecophysiology, which we refer to as Pathogen and Insect Pathways (PIPs). By varying the magnitude of ecophysiological impacts within PIPs, ecosystem models can predict the timing and magnitude of growth reductions and mortality, as well as subsequent impacts on community dynamics, biogeochemistry and biophysics, in a way that is more realistic than instantaneous mortality in response to FIPs. In the following sections we describe the proposed scheme in detail. We then use a simple ecophysiological model to demonstrate how this scheme generates predictions of growth reductions and mortality rates that are consistent with observed impacts. Finally, we show that the proposed scheme makes novel predictions about feedbacks between FIP impacts and plant water use, allocation and turnover.

## Conceptual model

**Figure 1:**
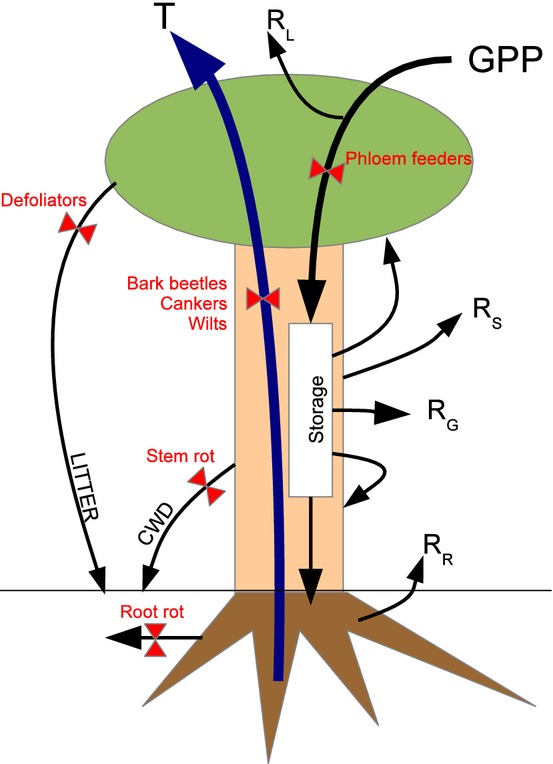
A general model for incorporating biotic disturbance in ecosystem models. A typical ecosystem model predicts GPP based on enzyme kinetics or light-use efficiency and is closely coupled to transpiration through stomatal regulation. Plants then allocate stored carbon to leaf, root and stem pools minus construction costs (growth respiration, R_g_). Maintenance respiration (R_l_, R_s_ and R_r_) and turnover then deplete each of these pools to CO_2_ and soil organic matter respectively. Biotic disturbances (red font) act by altering one or more of these flux terms (red valves): For leaves and roots this is through a percent (%) acceleration of turnover rates; for sapwood a % hydraulic reduction; for heartwood a % loss of physical strength and for storage a % loss of inputs.

The proposed approach classifies biotic disturbance agents into five PIPs based on their physiological impacts on the three major biomass pools (root, leaf, stem) and two transport tissues (xylem, phloem) in trees (Fig. 1). This approach is extensible to a wide range of ecosystem models, as most models share this basic representation of plant physiology. The central concept of this scheme is to parameterise different insects and pathogens based on their direct physiological effects through the PIPs functional approach, and then predict subsequent impacts on demography, succession, biogeochemistry and land surface biophysics. The intensity of any particular PIP is conditional on management actions and insect and pathogen dispersal, colonisation and proliferation, which would be estimated outside of the ecosystem model using integrated assessment approaches (Ibanez *et al.* 2014), though there are obviously significant uncertainties in such projections.

Under this scheme different FIPs are classified functionally into five groups: defoliators, root rots, stem rots, xylem reducers and phloem feeders (Table 1). For example, insect defoliators, such as spruce budworm (*Choristoneura*), Gypsy moth (*Lymatria dispar*), and forest tent caterpillar (*Malascoma disstria*) are in the same PIP as the fungal pathogen Swiss needle cast (*Phaeocryptopus gäumannii*) and modelled based on the proportion of leaf biomass removed. Root rots, root-feeding weevils and parasitic nematodes are similarly modelled by the proportional increase in root turnover rate. Stem rots increase the background mortality rate, which is the equivalent to an increase in the stem turnover rate. In terms of impacts of FIPs on transport, bark beetles and wilts would both be modelled by a proportional reduction in xylem transport. Specifically, most models include a mechanism that allows soil moisture availability to reduce transpiration below that predicted by leaf-level stomatal conductance (De Kauwe *et al.* 2013). This PIP would reduce the soil moisture ‘supply’ capacity. Phloem feeders, such as hemlock woolly adelgid (*Adelges tsugae*), would remove a fraction of the non-structural carbon exported from leaves. Finally, this generalised ecophysiological PIPs framework allows a single insect or pathogen to act through multiple pathways. For example, a stem rot that causes the loss of whole branches is also acting as a defoliator.

**Table 1:** Pathogen and insect pathways

Functional group	Impact	Examples
Phloem	% Carbon intercepted	Hemlock Woolly Adelgid (*Adelges tsugae*)
Xylem	% Reduction in water supply rate	Bark beetle (subfamily Scolytinae)
Leaf	% Removal	Gypsy moth (*Lymantria dispar*)
Root	Increase in turnover rate	Root rot, weevils (superfamily Curculionoidea)
Stem	Increase in per-capita mortality	Stem rot

See Hicke *et al.* (2012, tables 1 and 2) for descriptions of impacts of example and additional species.

## Methods

To evaluate the ability of the proposed approach to simulate realistic ecosystem changes as a result of FIPs, we implemented the PIP functional groups into a simple ecophysiology-based stand model. This model was run through a series of 4-year simulations to evaluate the cascading, multi-year effects of FIPs on growth and survival. The model was forced with 30-minute meteorology from the Metolius intermediate aged ponderosa pine (US-Me2) Ameriflux tower, located in central Oregon (44.4523N, -121.5574E; http://ameriflux.ornl.gov/). Meteorology for the year 2005 was recycled to eliminate the effects of interannual variability from the modelled responses and focus instead on the impact of FIPs on ecosystem processes. Mean annual temperature was 7.3°C and the site received 550 mm of precipitation in 2005. Simulations assumed an even-aged (10 cm DBH) temperate evergreen conifer stand with 700 trees/ha, similar to the structure observed at this site (Youngblood *et al.* 2004). By using an even-aged evergreen species in this initial proof-of-concept example, we avoided the complicating effects of phenology and canopy heterogeneity.

For each PIP, we evaluated the sensitivity of tree biomass pools and stem density in response to varying PIP severity across its allowable range of ecophysiological impact. The exploratory model took a simplified cohort-based approach where the fundamental state variables were the stand-level stem density and the tree-level leaf, stem, root and storage carbon pools. The model was implemented in R and the full code is available at https://github.com/mdietze/PestED. Model structure (Fig. 1) is based loosely on the Ecosystem Demography model (Medvigy *et al.* 2009) and is summarised below with the representation of each PIP described when the relevant ecophysiological process is introduced. Model parameters are in Table S1 (Supporting Information).

Leaf-level photosynthesis was modelled using a standard enzyme-kinetic approach (Farquhar *et al.* 1980) coupled to the Medlyn variant of the Ball–Berry stomatal conductance model (Medlyn *et al.* 2011) and scaled to gross primary productivity (GPP) based on leaf area index (LAI). Photosynthetic parameters were assumed to vary with temperature according to Arrhenius functions as used in (Dietze 2014). Water supply was calculated as proportional to soil water and fine root biomass. Soil water was approximated with a bucket model with inflows of precipitation and outputs of transpiration and soil evaporation. GPP and transpiration (T) were reduced linearly if the water supply rate fell below the water demand calculated from stomatal conductance and the vapour pressure deficit (VPD). Leaf and canopy boundary layer conductance were assumed to be negligible. As noted above, the xylem-disrupting PIP acts through a fractional reduction in water supply rate.

Leaf (R_L_), fine root (R_R_) and stem (R_S_) respiration were assumed to be proportional to these biomass pools and to vary as an Arrhenius function of temperature. Carbon exported from leaves (GPP – leaf respiration) was first transported to a storage biomass pool. This phloem flux was taxed by the phloem PIP as a fractional removal of net carbon gain. The stem biomass was modelled as an empirical allometric function of DBH. The capacity of the leaf biomass pool was also modelled with an empirical allometry, though the realised leaf biomass depended upon the balance between leaf allocation and leaf turnover. The defoliator PIP was implemented as a single, instantaneous removal of some fraction of leaf biomass. The capacities of the fine root and storage biomass pools were modelled as fixed ratios to the capacity of the leaf biomass pool. As with leaf biomass, the realised fine root biomass depended upon the balance between root allocation and root turnover. The root PIP was implemented as a proportional increase in root turnover rate. Soil organic matter (SOM) increases due to inputs from leaf and root turnover as well as whole tree mortality. Soil heterotrophic respiration, which decreases SOM, is assumed to be proportional to pool size with a Q10 temperature sensitivity.

The realised storage biomass pool depended upon the inputs from the phloem and exports to allocation. There was no additional turnover or respiration for the storage pool, but allocation from storage to new growth incurred a growth respiration (R_G_) cost. The allocation out of the storage pool occurred according to the following rules and priorities (Waring 1987; Dietze *et al.* 2014).

1. Carbon is only available once it is in the phloem.
2. Carbon is first used to meet the demands of maintenance respiration.
3. Carbon is then allocated to storage until the plant has enough carbon to meet the maintenance respiration demands for K days.
4. Allocation next brings leaf and root pools up to a specified minimum fraction of their capacity.
5. Allocation is then used to fill the storage pool.
6. Leaf and root pools are next brought to their allometric capacity.
7. Any available carbon is then split between stem growth and reproductive output according to a fixed fraction. Allocation to stem increases the DBH allometrically and thus increases the capacities of the leaf, fine root and storage pools. Allocation to reproduction is assumed to increase stem density.

In this test case an evergreen phenology was assumed to avoid complicating allocation rules with phenology in this proof-of-concept modelling experiment. Finally, tree mortality probability is assumed to vary as a negative exponential function of the filled fraction of total storage capacity. This implies that, on average, trees with a full storage pool have a lower probability of mortality than trees where that storage pool is depleted (Kobe 1997; Canham *et al.* 1999; Myers & Kitajima 2007; Galiano *et al.* 2011). Given the allocation priorities above, this relationship is also consistent with observations of a negative exponential relationship between diameter increment and mortality probability (Kobe *et al.* 1995; Wyckoff & Clark 2000). Tree mortality reduces stem density but does not change the size of the tree-level leaf, root and stem biomass pools. The stem PIP is implemented as an additive increase in the mortality rate.

## Results

**Figure 2:**
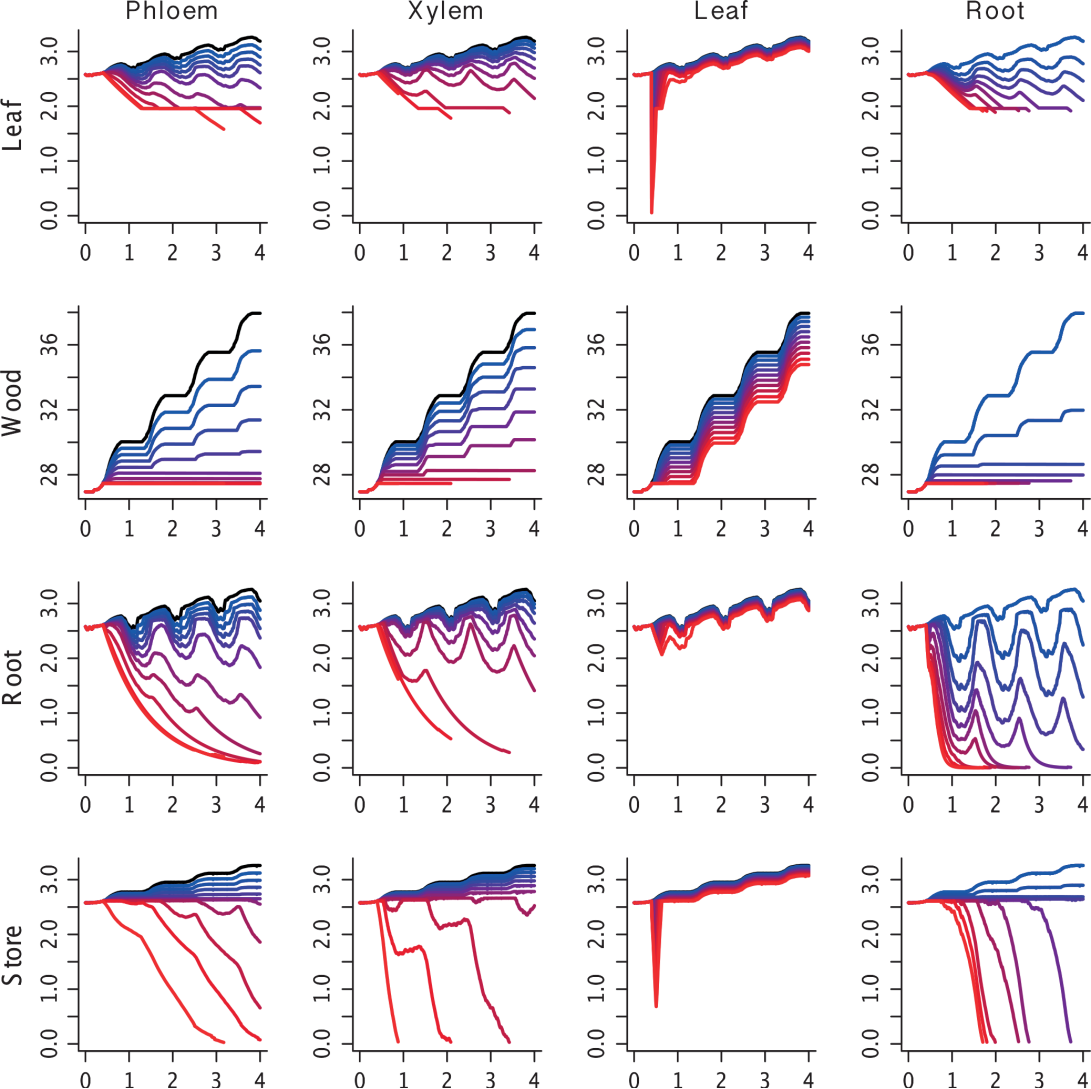
Impact of biotic disturbance on biomass pools (kg/plant) arranged by PIP (columns) and pool (rows) over a 4 year period. For phloem, xylem and leaf, each line is associated with an additional 10% loss ranging arranged from the lowest (dark blue, 10%) to the largest (red, 90%), whereas for roots the same gradient represents an increase in turnover rate by 1× (dark blue) through 9× (red). Default run is in black. It is important to note that mortality does NOT reduce sizes of individual's biomass pools, but rather the stem density.

**Figure 3:**
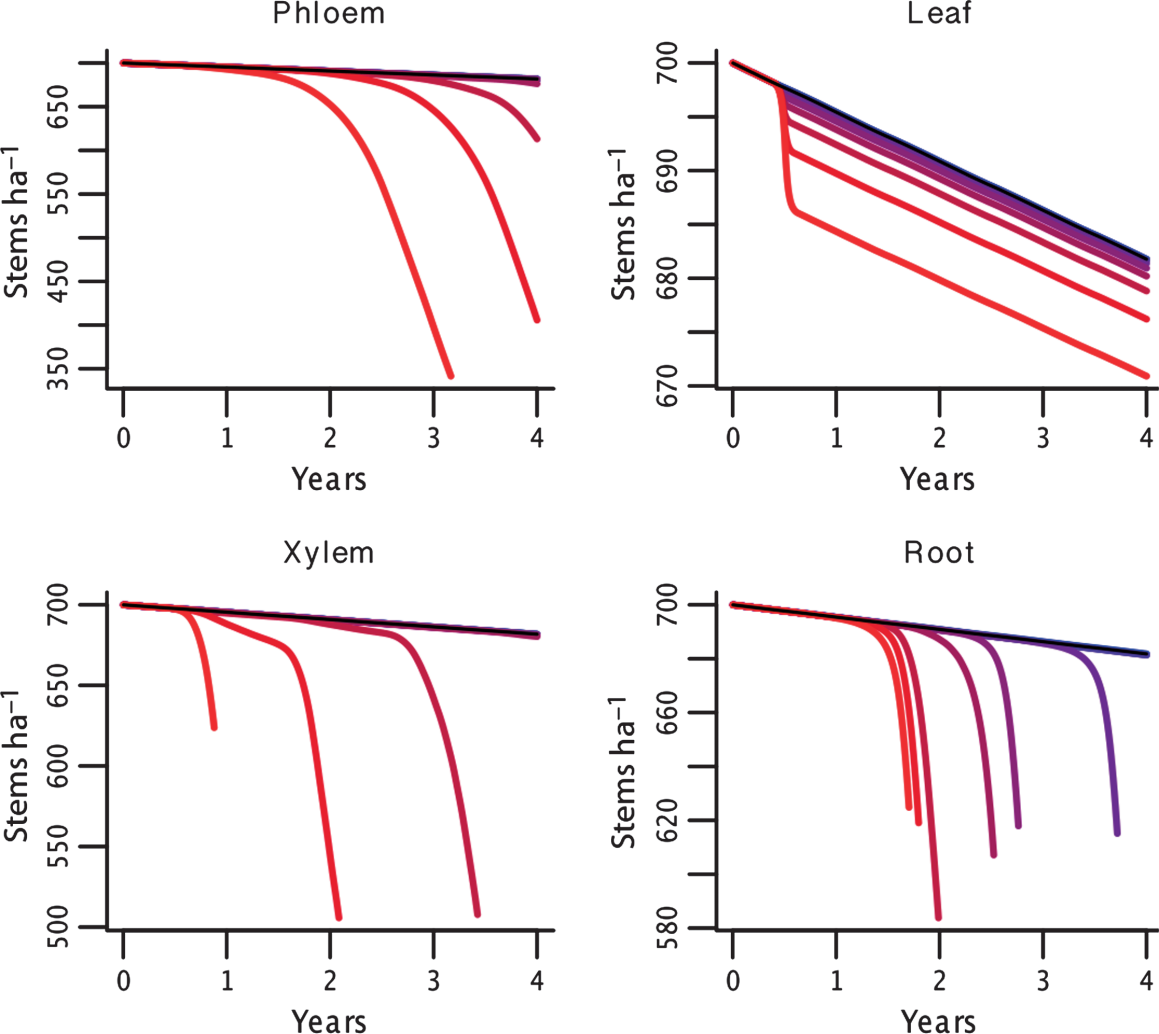
Mortality in response to biotic disturbance. Change in stem density through time (years) as a function of disturbance intensity. Intensity levels are as described in Figure 2. Note that the range of densities observed varies considerably among the four cases. Also the cessation of a time series at a point before the end of 4 years is associated with a complete stand-level die-off at that point in time, with stem density going to zero. Finally, in all cases there is the identical level of background mortality occurring in the absence of biotic disturbance.

**Figure 4:**
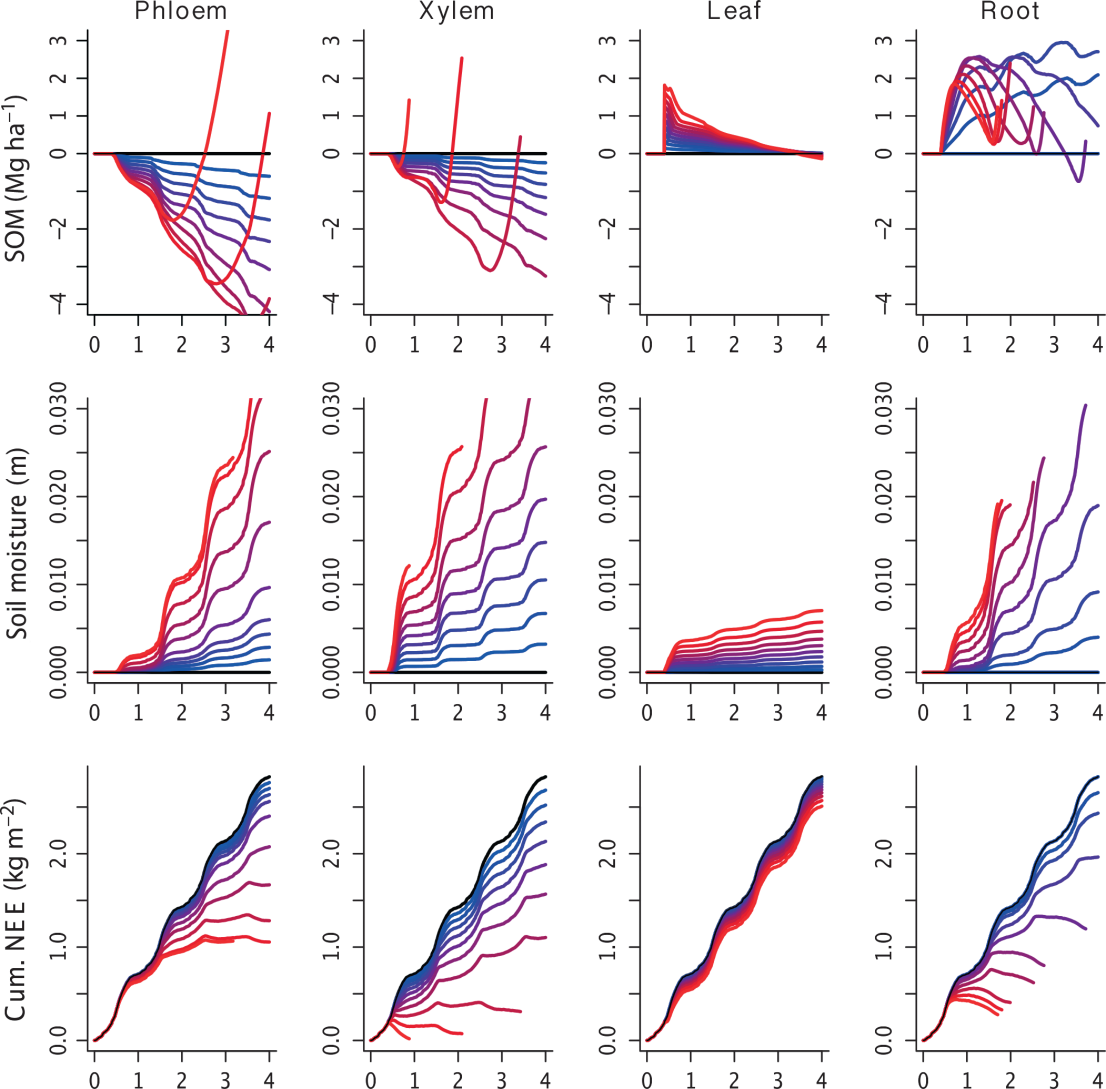
Ecosystem responses to biotic disturbance over a 4 year period as a function of disturbance intensity. Impacts are arranged by PIP (columns) and intensity levels are as described in Figure 2. Soil organic matter (SOM) and soil moisture are expressed as the deviation from the default run (black line at 0) with negative indicating a loss, whereas the net ecosystem exchange of carbon (NEE) is expresses as the cumulative over the 4 year period.

Implementing the PIPs functional framework into a simple ecosystem model reproduced a wide range of realistic responses in tree biomass pools (Fig. 2), stem density (mortality rates, Fig. 3) and ecosystem fluxes (Fig. 4). More sophisticated modelling will be required to understand the long-term feedbacks of elevated mortality on stand succession, biogeochemistry and biophysics, but this framework is a critical first step towards simulating the realistic and generalised impacts of FIPs within forest ecosystems. The results for the stem PIP are not discussed further, as the short-term impacts of this PIP in the simple ecosystem model are simply a reduction in stand-level stem density without an appreciable change in biomass pools.

### Phloem transport

At low to moderate levels of phloem feeding, trees showed modest reductions in leaf, root and storage pools, but a disproportionate reduction in wood growth (i.e. a 10% reduction in phloem caused a 20% reduction in growth). This decrease was slightly smaller with each additional reduction, where growth declined an additional 19, 18 and 12% as phloem transport was, respectively, reduced to 20, 30 and 40%. A 50% reduction in phloem transport caused a cessation of new woody growth, after which additional phloem feeding began to have large impacts on leaf, root and storage pools. That said, even at high levels of stress, tree mortality tended to be a gradual, delayed process. Over the range of phloem feeding that resulted in growth reductions, the ecosystem-level response was a modest reduction in SOM, a modest increase in soil moisture and a fairly small change in cumulative net ecosystem exchange (NEE). However, over the range of disturbance intensities that triggered stand-level die-off there was a rapid increase in SOM due to the large inputs of coarse woody debris, much larger increases in soil moisture and a strong reduction in NEE.

### Xylem transport

In contrast to phloem feeders, where losses created a slight decline in growth and biomass pools, the impacts of xylem loss were initially modest (a 10% reduction in xylem caused a 10% reduction in woody growth) but increased with each additional reduction in xylem transport. Specifically, additional xylem reductions at 20–70% cause growth reductions of 11, 12, 13, 15 and 17%. At the highest levels of infection, xylem loss induced the most rapid stand-level mortality among the five PIPs. The ecosystem-level response to xylem reduction was similar to that observed in response to phloem reduction, where growth reductions resulted in a decrease in SOM, increase in soil moisture and reduction in NEE. NEE was noticeably more sensitive to xylem reduction than to phloem feeding and at the most severe intensities stands became a net carbon source even before die-off commenced. Also, the effect of xylem reduction on soil moisture was immediate, leading to increases in the same growing season as the initial infection, whereas for phloem feeding the soil moisture response was minor in year one but became more pronounced in the second growing season.

### Defoliation

Foliar losses caused an approximately linear reduction in woody growth in the current growing season. At the highest levels of defoliation the losses to biomass pools carried over to the next year. Mortality was elevated over background rates, from around 0.5% to 2.5%, but this increase was limited to the growing season in which defoliation occurred. Overall, for healthy trees exposed to a single defoliation event, this PIP had the lowest impact. That said, there is considerable potential for larger impacts on already stressed trees when faced with repeated defoliation events, or for interactions between defoliators and other biotic and abiotic disturbances. At the ecosystem-level, defoliation caused a net increase in SOM, but this effect decayed over time and all treatments converged back to the control within 3 years. The defoliation also resulted in an increase in soil moisture, but more surprisingly this effect did not decay over time and indeed increased slightly over time. Finally, NEE declined slightly in response to defoliation but this effect was largely limited to the year of defoliation, with very minor legacy effects due to increased litter.

### Root turnover

In cases of large amounts of root loss, woody growth reductions were severe and exhausted tree carbon stores. Mortality from root rot was elevated from the control background rates over a wide range of severities and in general occurred at an intermediate rate between phloem and xylem loss. At the ecosystem-scale, root loss caused an immediate increase in soil moisture, though with a lower sensitivity than observed for xylem reduction. SOM had a complex response to root loss, with low levels of loss leading to a gradual increase in SOM, but with moderate to severe root loss leading to a rapid initial increase that is followed by a transient decline as these roots decay, and then a second increase in SOM as stand mortality occurs. Finally, NEE showed a high sensitivity to root loss that was driven by both increases in SOM and decreases in GPP.

## Discussion

One of the characteristic features of any severe forest disturbance is the large-scale mortality of trees, and for most of the PIPs considered, high PIP intensity produced stand-scale die-offs (Fig. 3). More importantly, mortality was never instantaneous under any PIP, which is an important difference between biotic disturbance and physical disturbances such as fire and windthrow. However, even in these abiotic cases resprouting is common, and the actual mortality of downed trees can be a drawn-out process (Dietze & Clark 2008). Furthermore, the timing of mortality across different PIPs is suggestive of the differences observed among different insects and pathogens. For example, the most rapid mortality was observed with the disruption of xylem transport, which is similar to the patterns observed in bark beetle outbreaks (Wullschleger *et al.* 2004; Kurz *et al.* 2008a). In contrast, the slow mortality within the phloem transport PIP is similar to the delayed mortality observed for hemlock woolly adelgid (Orwig & Foster 1998). Rates of mortality as a result of the defoliator PIP were also realistically modest, as single defoliation events rarely kill trees, but repeated events or defoliation of already stressed trees can trigger mortality (Cook *et al.* 2008; Clark *et al.* 2012). High mortality from root rot is generally not widespread in adult trees except in isolated pockets (Bloomberg & Reynolds 1985; Hansen & Michaels Goheen 2000), so the high sensitivity predicted by this model suggests that most infections within ecosystems are of low severity or limited spatial extent. In contrast, this mortality pathway is quite common in seedlings and is thought to be a major source of biodiversity-stabilising density dependent mortality (Bagchi *et al.* 2014). The ability of this simple model to accurately reproduce realistic patterns of mortality prompted by diverse FIPs lends credence to the modelling approach proposed here.

In addition to the direct effect of each of the five PIPs on tree physiology, the model demonstrated the potential for interactions between water stress and biotic infections. This feedback is understandably most dramatic for root diseases, where the direct loss of roots reduces water supply and thus reduces the supply of new carbon available to replace lost roots. However, there is also evidence for root declines in the xylem-disrupting PIP, where water limitation likewise reduces GPP, and in the phloem feeder strategy, where GPP is intercepted by FIPs. In both instances, root biomass declines further in response to the decrease in GPP instigated by PIPs. In all cases, if hydraulic failure had been implemented as a source of mortality it is likely that these feedbacks would have been even stronger. It is straightforward to extend the proposed scheme to mortality from hydraulic causes and, rather than being a counter example, this is completely consistent with the hypothesis we put forth: that the primary effects of FIPs can be captured based on their direct impacts on transport and turnover. Overall such feedbacks are indicative of the classic idea of a tree mortality ‘death spiral’ (Franklin *et al.* 1987), where one stress increases another and the final cause of death may be very different from the initial trigger. Alternatively, if foliage is lost at a greater rate than fine roots then soil moisture will increase due to reduced transpiration, whereas the soil surface may dry out from increased evaporation. The ability of the PIPs framework to capture these alternative feedbacks highlights the importance of including these mechanisms within ecosystem models.

While the model exhibited negative feedbacks with declines in leaf and fine root biomass in response to moderate or severe biotic infection, the most common response to mild infection was a woody growth reduction. This response was a direct result of the modelling assumption that woody growth has a lower allocation priority than leaves and fine roots, but that assumption is consistent with a wide range of observations and experiments (Kozlowski 1992; Dietze *et al.* 2014). Given the potential for feedbacks due to leaf or fine root loss, this prioritisation is also a sensible strategy. While alternative allocation schemes were not implemented in this simple model, it seems clear that any scheme that gave woody growth a higher priority would accelerate the death spiral at moderate to severe levels of infection.

Another key result from implementing the PIPs scheme within this simple ecosystem model is that the different PIPs clearly varied in their growth sensitivity (Fig. 2). Defoliation had the most linear response of the PIPs, with close to a 1 : 1 response between foliage removal and growth reduction, whereas phloem feeding had a higher sensitivity, closer to a 2 : 1 response. Xylem loss had a modest impact on growth at low levels, which is sensible since trees are likely operating below their maximum conductance under average conditions. However, each additional level of xylem loss led to a progressively larger, nonlinear, impact on growth. It should be noted that the current model assumes an isohydric response, whereby stomata close in response to water deficit, and that the response to xylem loss is likely to change somewhat in anisohydric species, where xylem loss would instead increase the risk of cavitation (Tardieu & Simonneau 1998). Finally, root rot had the highest growth sensitivity, effectively ceasing growth in response to a 3× increase in turnover rate.

In all the above simulations we assumed that biotic agents acted through a single pathway on an unstressed individual. In the general framework we have proposed it would be straightforward to represent biotic disturbances that act through multiple pathways simultaneously, for example by disrupting either xylem and phloem transport or causing both root and stem rot. In addition, we are likely to see more dramatic growth and mortality responses in individuals suffering from multiple stresses simultaneously, such as drought and bark beetles (Breshears *et al.* 2005), or which are recovering from previous stress, such as repeated defoliation (Campbell & Sloan 1977).

Moving forward, while the results of these simulations are qualitatively consistent with observed biotic disturbances, there is a need to evaluate this approach for a range of case studies to determine how the proposed scheme works in practice. In particular, we need to test whether the proposed ecophysiological impacts alone are sufficient to reproduce quantitative patterns. Alternatively, there could be feedbacks not represented by the current scheme that either accelerates mortality, such as nonlinearities due to multiple concurrent stressors, or feedbacks that slow down rates of infection, such as mobilisation of plant defences. Furthermore, the proposed scheme needs to be implemented in more sophisticated terrestrial biosphere models to more fully explore the impacts of growth and mortality on vegetation dynamics, biogeochemistry, hydrology and the surface energy budget and to compare these predictions to observations. In particular, the successional trajectory of a stand will respond not only to the scale and extent of die-off but also the delayed pace of these die-offs compared to physical disturbances. All else being equal, delayed die-off will favour recruitment of advanced regeneration already in the understory over shade-intolerant pioneers. Finally, the proposed scheme has the potential to make novel predictions of FIP responses under future scenarios. This allows us to evaluate the interactions between biotic disturbances and the ecophysiological impacts already represented in the current generation of models, including but not limited to changes in climate, extreme weather, elevated CO_2_, ozone and nitrogen deposition.
