# Supplementary material for: A general ecophysiological framework for modelling the impact of pests and pathogens on forest ecosystems
Source: Ecol Lett. 2014 Aug 28;17(11):1418–26. doi: 10.1111/ele.12345 (PMC4257091; doi:10.1111/ele.12345)
Supplement: Supplementary file 1 — Supplementary [file ele0017-1418-SD1.docx]

Table 1: Model Parameters

| Name | Value | Description |
| --- | --- | --- |
| gevap | 5.00E-03 | Soil/Air conductivity (m^2^/s) |
| Wthresh | 1 | Threshold in plant available moisture trapezoidal response (m^3^/m^2^) |
| Kroot | .2 | Water uptake per kg root per m available water |
| SLA | 10 | Specific Leaf Area (m^2^/kg) |
| alpha | 0.8 | Quantum yield |
| Vcmax | 18 | Maximum carboxylation rate (μmol/m^2^/s) |
| Jmax | 30 | Maximum electron transport rate (μmol/m^2^/s) |
| m | 4 | Stomatal slope |
| g0 | 0 | Cuticular conductance |
| allomB0 | 9.90E-02 | Stem biomass allometry: intercept |
| allomB1 | 2.43 | Stem biomass allometry: slope |
| allomL0 | 5.46E-02 | Leaf biomass allometry: intercept |
| allomL1 | 1.67 | Leaf biomass allometry: slope |
| Rleaf | .72 | Basal leaf respiration (μmol/m^2^/s) |
| Rroot | 1.2 | Basal root respiration (μmol/kg/s) |
| Rstem | 0.05 | Basal stem respiration (μmol/kg/s) |
| Rg | .33 | Growth respiration fraction |
| leafLitter | 1.88E-05 | Leaf turnover, per 30 min timestep |
| CWD | 5.71E-09 | Stem turnover, per timestep |
| rootLitter | 5.71E-05 | Root turnover, per timestep |
| mort1 | 1 | Mortality rate at zero storage, year^-1^ |
| mort2 | 5 | Decay constant in mortality/storage relation |
| NSCthreshold | 0.01 | Fraction of Smax at which cohorts are removed |
| Lmin | .75 | Fraction of leaf biomass above which storage occurs |
| q | 1 | Leaf:root biomass ratio |
| StoreMinDay | 2 | Minimum number of days worth of respiratory carbon that must be kept as Storage |
| Smax | 1 | Storage capacity to leaf biomass ratio |
| Rfrac | .2 | Fraction of available C used for reproduction |
| SeedlingMort | .99 | Seedling mortality rate |
| Kleaf | 1.75E-04 | Leaf growth rate at 10 °C, proportion per timestep |
